# Web-based high-intensity bodyweight interval training improves metabolic health and physical fitness outcomes in middle-aged men with obesity during the COVID-19 pandemic

**DOI:** 10.3389/fphys.2025.1711436

**Published:** 2025-11-24

**Authors:** Dong-Joo Hwang, Dong-Hun Choi, Ah-Hyun Hyun

**Affiliations:** 1 Exercise Biochemistry Laboratory, Korea National Sport University, Seoul, Republic of Korea; 2 Sport Science Institute, Korea National Sport University, Seoul, Republic of Korea; 3 Department of Sports Medicine, Konyang University, Nonsan, Republic of Korea; 4 Department of Military Kinesiology, Korea Military Academy, Seoul, Republic of Korea

**Keywords:** high-intensity interval training (HIIT), web-based exercise intervention, obesity, metabolic health, COVID-19

## Abstract

Reduced opportunities for physical activity during the COVID-19 pandemic have heightened obesity-related health risks, emphasizing the need for effective, scalable, and remotely deliverable exercise interventions. This randomized controlled trial examined the efficacy of an 8-week, real-time, supervised, web-based high-intensity interval training (HIIT) program in improving metabolic health and physical fitness in middle-aged men with obesity. Twenty-two men (age <45 years; BMI >30 kg/m^2^; waist circumference >90 cm) were assigned to a videoconference-based HIIT intervention or a non-exercise control group. The HIIT group completed two supervised sessions per week, each consisting of a standardized warm-up, a 20-min HIIT protocol, and a cool-down, with real-time monitoring and weekly dietary log reviews. Web-based HIIT significantly reduced fat mass by 6.6% (−2.01 kg; *d* = 1.38) without altering total body weight and improved lipid profiles by increasing HDL cholesterol and decreasing total and LDL cholesterol. Leptin levels decreased, adiponectin increased, and IL-10 rose, whereas IL-6 remained unchanged. Cardiorespiratory fitness improved, with VO_2_max increasing by 3.06 mL·kg^-1^·min^-1^ (∼8.7%) and minute ventilation increasing, and muscle performance was enhanced, as trunk extensor peak torque and average power increased by 9.7% and 30.2%, respectively, and knee flexor peak torque increased by 31.8% (right) and 19.5% (left), yielding large effect sizes (*d* = 0.9–1.3). These findings indicate that real-time, non-face-to-face HIIT effectively enhances body composition, lipid metabolism, inflammatory balance, aerobic capacity, and functional muscle performance in men with obesity, supporting this contactless modality as a feasible and deployable strategy for health maintenance during and beyond pandemic-related restrictions.

## 1 Introduction

The COVID-19 pandemic drastically reduced opportunities for physical activity due to social distancing and the closure of exercise facilities, resulting in a 40%–60% decline in exercise participation and a rapid increase in obesity among men (Luzi and Radaelli, 2020; Castañeda-Babarro et al., 2020; Furtado et al., 2021). The widespread adoption of remote work further contributed to prolonged sedentary behavior and unhealthy lifestyle habits such as excessive alcohol consumption, late-night snacking, and smoking, which exacerbated metabolic health risks in men (Salvadori et al., 2021; Ricketts et al., 2016). Under these conditions, home-based bodyweight training emerged as a practical alternative for maintaining physical activity during lockdowns (Steele et al., 2021). The Centers for Disease Control and Prevention (CDC) highlighted the risks of physical inactivity during the pandemic and emphasized the importance of individual-level efforts to remain active; however, strict environmental restrictions made it particularly difficult to mitigate pandemic-induced obesity (Kraus et al., 2019).

Obesity is strongly associated with chronic systemic inflammation, leading to serious complications such as hypertension, diabetes, cardiovascular disease, and cancer (Gao et al., 2022; Gerosa-Neto et al., 2020). In particular, visceral fat accumulation resulting from physical inactivity elevates pro-inflammatory cytokine expression, and obesity has been closely linked to COVID-19 mortality. In particular, visceral fat accumulation resulting from physical inactivity elevates pro-inflammatory cytokine expression, and obesity has been closely linked to adverse COVID-19 outcomes, including higher rates of hospitalization, severe disease, and mortality. A meta-analysis of >400,000 patients reported that individuals with obesity were 2.3 times more likely to be hospitalized and 2.6 times more likely to die from COVID-19 compared with those with normal weight (Popkin et al., 2020; Kompaniyets et al., 2021).

Cytokines secreted from adipose tissue, such as IL-6 and CRP, are typically elevated in abdominal obesity (Favre et al., 2021), and excessive cytokine expression may promote macrophage activation and oxidative stress, leading to cellular and DNA damage (Chiappetta et al., 2020). Anti-inflammatory mediators such as IL-10 play a compensatory role but are usually reduced in individuals with obesity (Codella et al., 2021). Previous studies have reported that IL-10 levels are negatively correlated with insulin resistance and positively correlated with insulin sensitivity, suggesting that higher IL-10 concentrations contribute to improved metabolic regulation in individuals with obesity (da Silveira et al., 2021). Lower muscle mass further contributes to heightened inflammation and metabolic dysfunction (Trinity et al., 2021).

Leptin and adiponectin, two major adipokines regulating energy metabolism, are key determinants of metabolic health and disease severity in obesity (Di Filippo et al., 2021). Leptin resistance arising from chronic overnutrition blunts appetite control and fat metabolism (Montserrat-De La Paz et al., 2021; Dey et al., 2021), while reduced adiponectin impairs glucose regulation and insulin sensitivity (Xu et al., 2020; Ahl et al., 2015). This hormonal imbalance contributes to insulin resistance and increases the risk of severe COVID-19 outcomes (Kwok et al., 2020). Therefore, lifestyle-based interventions that reduce fat mass and restore metabolic balance remain essential during restricted living conditions.

The World Health Organization (WHO) and the American College of Sports Medicine (ACSM) recommend at least 150 min of moderate-intensity or 75 min of vigorous-intensity exercise per week (World Health Organization, 2020; Denay et al., 2020). However, pandemic restrictions have highlighted the value of non–face-to-face, web-based exercise programs as feasible and scalable alternatives for maintaining physical activity (de Oliveira Neto et al., 2020). Among these, bodyweight-based interval training (a form of weight-bearing exercise using one’s own body mass) has gained attention as a practical strategy for eliciting vigorous-intensity responses comparable to conventional high-intensity interval training (HIIT) modalities such as cycling or treadmill running (Li et al., 2023; Schaun et al., 2018; Bellissimo et al., 2025; Hortmann et al., 2021). Men, in particular, tend to prefer dynamic and resistance-oriented exercise modalities that engage large muscle groups and emphasize strength and power components, making bodyweight-based programs an appealing and accessible option (Chatfield et al., 2018; Oyibo and Vassileva, 2020). This type of training can be flexibly tailored through exercise selection (e.g., squats, lunges, push-ups, burpees), work-to-rest ratios, and progressive intensity adjustment, allowing individualized control over load and recovery even in home settings (Li et al., 2023). This approach involves alternating bouts of vigorous exercise and recovery, enabling substantial improvements in metabolic, cardiovascular, and muscular outcomes. Previous studies, including several meta-analyses, have consistently reported that HIIT is more effective than moderate-intensity continuous training (MICT) in reducing total and visceral fat mass, improving VO_2_max, and enhancing insulin sensitivity (Da Silva et al., 2020; Motiani et al., 2019; Hyun, 2021; Keating et al., 2017; Milanović et al., 2015; Jelleyman et al., 2015). A recent study reported that web-based HIIT safely improves physiological parameters and physical fitness in abdominally obese women (Hyun, 2021). However, although several web-based exercise interventions have targeted women and older adults, comparatively fewer randomized trials have focused solely on men with obesity (Dulin et al., 2023; Keshavarz et al., 2023). Accordingly, the development and validation of real-time, web-based bodyweight-interval training (BW-IT) for men with obesity are urgently required.

Maintaining regular exercise during social isolation is crucial for preserving both physical and mental health. Although web-based interventions have been shown to increase physical activity (Zhang et al., 2021; Schwendinger and Pocecco, 2020), their effects on obesity-related biomarkers and physical fitness in men are not well established. Given its accessibility, cost-effectiveness, and scalability, real-time web-based BW-IT may serve as a practical strategy to improve metabolic health and physical performance during and beyond pandemic restrictions (Thomson et al., 2023; Lewis et al., 2023). Therefore, this study aimed to investigate the effects of real-time, web-based bodyweight interval training on obesity-related indicators, metabolic health, and physical fitness in middle-aged men with obesity.

## 2 Materials and methods

### 2.1 Participants

**FIGURE 1:**
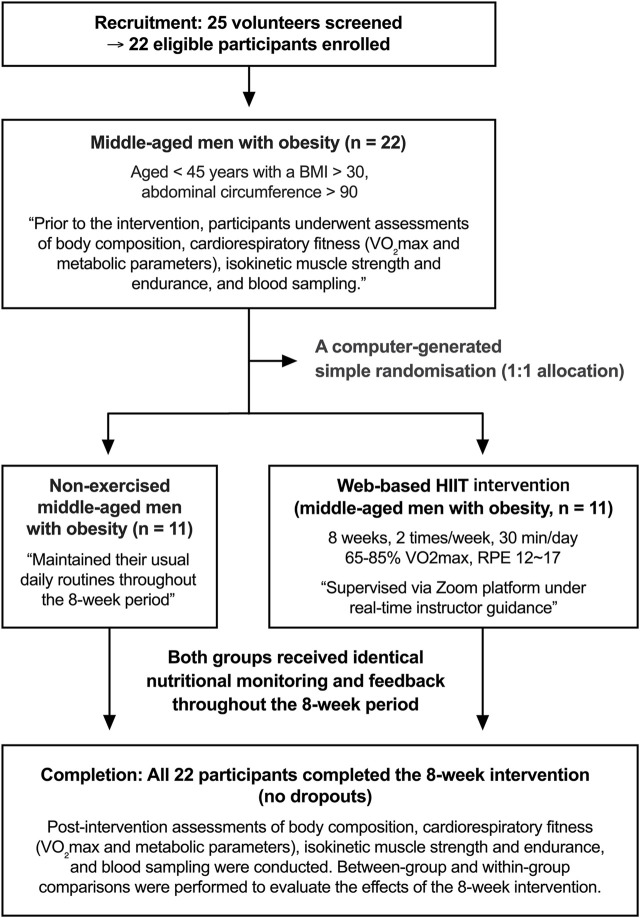
Flowchart illustrating the study design and intervention protocol.

The subjects of this study were men under the age of 45 years with a body mass index (BMI) > 30 kg/m^2^ and an abdominal circumference >90 cm. A total of 22 men with obesity were recruited through an online community and provided written consent prior to enrollment. The consent process included information about the study purpose, procedures, potential risks, and the right to withdraw at any time (no minors were included in the study). Eligibility was confirmed through a structured telephone interview conducted by a trained researcher, during which participants were asked about their exercise habits, medical history, and medication use to verify inclusion criteria. Eligible participants were randomly allocated to either the high-intensity interval training (HIIT) group (n = 11) or the control (CON) group (n = 11) using computer-generated simple randomization (1:1 allocation), as illustrated in Figure 1. The randomization sequence was created using a pseudo-random number generator by an independent researcher not involved in data collection or analysis. Allocation concealment was maintained until completion of baseline testing. No stratification or blocking was applied because of the small sample size, and while participants and exercise supervisors were aware of group assignments, outcome assessors and the statistician were blinded to allocation to minimize bias. This study was approved by the Institutional Ethics Committee of Korea National Sport University (KNSU) (KNSU-20210916-141) and conducted in accordance with the Declaration of Helsinki. The physical characteristics of subjects are summarized in Table 1.

**TABLE 1:** Characteristics of participants.

Group	Age (yr)	Height (cm)	Weight (kg)	Body fat (%)	Lean mass (kg)	BMI (kg/m^2^)
Overall (n = 12)	37.65 ± 3.02	176.45 ± 1.67	92.69 ± 5.33	32.98 ± 1.65	59.55 ± 2.95	30.20 ± 1.09
CON (n = 11)	34.9 ± 1.28	177.0 ± 1.73	92.13 ± 6.51	32.04 ± 1.58	59.9 ± 3.1	30.3 ± 1.14
HIIT (n = 11)	40.4 ± 0.91	175.9 ± 1.48	93.25 ± 4.07	33.91 ± 1.15	59.2 ± 2.9	30.1 ± 1.09

Values are presented as mean ± standard deviation (SD); BMI, body mass index.

### 2.2 Web-based HIIT intervention

In this study, participants in the exercise group completed an 8-week program delivered through a real-time videoconferencing platform (Zoom). Exercises were demonstrated on personal computers or mobile devices, and participants performed them simultaneously under live supervision. An exercise supervisor continuously monitored participants’ conditions during each session and instructed them to stop immediately if any discomfort occurred. The supervisor also provided real-time feedback and answered participants’ questions as needed. In addition, participants were asked to record all foods and beverages consumed each day using a structured food diary throughout the intervention period. A registered dietitian reviewed the diaries once per week, provided corrective feedback, and encouraged compliance with the 2020 Dietary Reference Intakes for Koreans (KDRIs) to ensure appropriate caloric and nutrient balance. This continuous dietary monitoring aimed to reduce inter-individual variability and to support consistent nutritional intake across participants.

The HIIT sessions were conducted twice weekly for 8 weeks. Each session included a 5-min warm-up, 20 min of main exercise, and a 5-min cool-down. Exercise intensity was maintained at a rating of perceived exertion (RPE) of 12–17 on the Borg Scale, corresponding to approximately 65%–85% of measured VO_2_max values obtained from the graded exercise test (GXT). Exercise intensity was continuously monitored using participants’ wearable devices (smartwatches) to ensure the target effort was achieved. To monitor relative exercise intensity during each session, oxygen consumption was estimated from running speed using the following predictive equation: *VO*_*2*_ *(mL·kg* ^*-1*^ *·min* ^*-1*^ *) = [running speed (m·min* ^*-1*^ *) × 0.2] + 3.5.* Exercise duration was adjusted to approximate an energy expenditure of about 500 kcal per session, based on estimated oxygen consumption, to standardize workloads. This calculation was used solely to monitor training intensity and did not replace the directly measured VO_2_max values obtained from the GXT.

Participants performed the interval circuit in the following progressive levels, each representing a distinct movement category and relative intensity:

- Level 1: Lower-body exercises (squats, lunges)
- Level 2: Core exercises (plank, crunches)
- Level 3: Back exercises (back extensions)
- Level 4: Upper-body exercises (push-ups)
- Level 5: Arm and shoulder exercises (triceps extensions, military press using resistance bands)

These levels reflected a progressive overload design, with increasing movement complexity and relative intensity every 2 weeks according to participants’ fitness levels and perceived exertion. This approach ensured that the training adhered to the defining HIIT principle of alternating vigorous-intensity effort and recovery, while allowing individualized adjustment based on tolerance and adaptation. The detailed web-based HIIT program is presented in Table 2.

**TABLE 2:** High-intensity interval training intervention.

Modes	Contents	Time (min)	Set and rest	RPE
Warm-up	Breathing, static stretching	5		12
Main exercise	Level 1: 1∼2 weeks	20	85% HRmax 30 s 60% hrmax. 30 s	15–17
	Squat and shoulder press. Push-up tap, side steps Lunge and spine twist, burpee test, legs raise, crunch			
	Level 2: 3∼4 weeks			
	Reverse lunge, squat jump, animal push up, side kick Plank, kick back, good-morning deadlift			
	Level 3: 5∼8 weeks		X 10	
	Hip hinge, wall squat, lunge-twist, high knee, plank Push-up and burpee jump, 100 breathing, side squat		Total 2 set	
Cool-down	Total body stretching	5		12

### 2.3 Body composition

For the body composition assessment, participants’ height (cm) and body weight (kg) were measured while wearing light clothing and in a fasting state (Dong-Sahn Jenix, Korea). Bone mineral density (BMD, g/cm^2^), body weight (kg), body fat percentage (%), lean mass (kg), fat mass (kg), and body mass index (BMI, kg/m^2^) were evaluated using dual-energy X-ray absorptiometry (DEXA; GX system, Madison, WI, United States). During the procedure, participants were asked to lie comfortably on the device, and continuous transverse imaging was performed for approximately 10 min.

### 2.4 Graded exercise test (GXT)

The treadmill graded exercise test (GXT) was performed in accordance with the guidelines of the American College of Sports Medicine (ACSM). Maximal oxygen uptake (VO_2_max), maximal heart rate (HRmax), minute ventilation (VE), respiratory exchange ratio (RER), and exercise duration were assessed during a maximal GXT using a computerized cardiopulmonary metabolic system (COSMED T170 DE treadmill and Quark CPET system; COSMED, Rome, Italy). Prior to each test, the metabolic system was calibrated according to the manufacturer’s guidelines. Participants completed a 3-min warm-up at 1.7 mph to familiarize themselves with the procedure, followed by a stepwise increase in treadmill velocity of approximately 0.8–0.9 mph every 3 min, consistent with the standard Bruce protocol.

### 2.5 Isokinetic strength and muscular endurance test

Isokinetic muscle strength and endurance of the trunk and lower extremities were assessed using a HUMAC NORM isokinetic dynamometer (CSMi, Stoughton, MA, United States). The range of motion was set from 0° of extension to 90° of flexion. Muscle strength was measured at an angular velocity of 60°/s over five repetitions, and muscular endurance was assessed at 240°/s over 15 repetitions. Prior to testing, participants completed three familiarization trials to ensure maximal effort during the assessments.

### 2.6 Blood collection and biochemical analyses

To analyze blood lipids (total cholesterol, HDL cholesterol, LDL cholesterol, and triglycerides), obesity-related hormones (insulin, leptin, adiponectin), and inflammation-related cytokines (IL-6 and IL-10), participants were instructed to fast for 12 h prior to testing. A 10 mL sample of venous blood was collected from the antecubital vein. Samples were allowed to clot at room temperature for 30 min and then centrifuged at 3000 rpm for 10 min to separate the serum. Serum samples were outsourced to a specialized clinical laboratory, the Green Cross Medical Foundation (Seoul, Korea), for analysis.

### 2.7 Statistics

Statistical analyses were performed using IBM SPSS Statistics, version 24.0 (IBM Corp., Armonk, NY, United States). Descriptive statistics are presented as means and standard deviations (SD). To evaluate the effects of the intervention, a two-way repeated-measures analysis of variance (ANOVA) with a 2 (group: HIIT vs. CON) × 2 (time: pre vs. post) design was conducted for each variable. When significant interactions were observed, simple-effects analyses were performed using paired t-tests to assess within-group changes over time and independent samples t-tests to compare between-group differences at each time point. Although the mean age differed slightly between groups (34.9 vs. 40.4 years), baseline comparisons confirmed that this difference was not statistically significant (*p* > 0.05). Random allocation and the within-subject nature of the repeated-measures design further minimized the potential influence of age-related confounding. Given the limited sample size and exploratory aim of this pilot trial, age was not included as a covariate to avoid model overfitting and unnecessary reduction of statistical power. Regarding multiplicity, no formal across-outcome correction for multiple comparisons (e.g., Bonferroni, Holm) was applied because tests targeted conceptually distinct physiological domains in this exploratory study; we therefore emphasize effect sizes and interpret p-values cautiously. Effect sizes were calculated and reported as partial eta squared (ηp^2^) for ANOVA and Cohen’s *d* (*d*) for within-group paired t-tests. According to established conventions, ηp^2^ values of 0.01, 0.06, and 0.14 were interpreted as small, medium, and large effects, respectively, while Cohen’s *d* values of 0.2, 0.5, and 0.80 were considered small, medium, and large. Statistical significance was set at p < 0.05.

## 3 Results

### 3.1 Subject changes in bone mineral density and body composition following HIIT

Body composition was evaluated to determine whether participation in the web-based HIIT program influenced weight-related and skeletal outcomes in middle-aged men with obesity. Table 3 summarizes the changes in bone mineral density (BMD) and body composition for the CON and HIIT groups. A significant group × time interaction was observed for fat mass (F (1, 20) = 7.081, *p* = 0.015, ηp^2^ = 0.261), indicating that changes over the intervention period differed between groups. Simple-effects analysis revealed a significant reduction in fat mass in the HIIT group (t (10) = 4.583, *p* = 0.001, *d* = 1.38), whereas no significant change was observed in the CON group. In contrast, no significant group × time interaction effects were found for BMD (F (1, 20) = 0.550, *p* = 0.467, ηp^2^ = 0.027), body weight (F (1, 20) = 1.947, *p* = 0.178, ηp^2^ = 0.089), percent body fat (F (1, 20) = 3.118, *p* = 0.093, ηp^2^ = 0.135), or lean mass (F (1, 20) = 0.394, *p* = 0.537, ηp^2^ = 0.019). These findings indicate that participation in the real-time, web-based HIIT program selectively reduced fat mass without inducing measurable changes in total body weight, lean tissue, or bone mineral density within the 8-week intervention period.

**TABLE 3:** Changes in bone mineral density and body composition following HIIT.

Body composition variable	Time point	CON (n = 11)	HIIT (n = 11)	Source of variation	F-statistic	*p*-value
BMD (g/cm2)	Pre Post	1.30 ± 0.02 1.29 ± 0.02	1.26 ± 0.02 1.27 ± 0.03	T	0.080	0.780
				G	0.860	0.365
				T × G	0.55	0.467
Body weight (kg)	Pre Post	92.13 ± 6.51 95.20 ± 3.96	93.25 ± 4.07 91.00 ± 3.17	T	0.047	0.830
				G	0.061	0.807
				T × G	1.947	0.178
Body fat (%)	Pre Post	32.04 ± 1.58 32.17 ± 1.69	33.91 ± 1.15 32.44 ± 1.15^#^	T	2.151	0.158
				G	0.302	0.589
				T × G	3.118	0.093
Lean mass (kg)	Pre Post	59.97 ± 3.14 61.81 ± 2.14	59.28 ± 2.90 59.32 ± 2.33	T	0.431	0.519
				G	0.161	0.693
				T × G	0.394	0.537
Fat mass (kg)	Pre Post	28.84 ± 2.83 30.47 ± 2.63	30.49 ± 1.68 28.48 ± 1.47^#^	T	0.075	0.787
				G	0.003	0.956
				T × G	7.081	0.015^*^

Two-way repeated-measures ANOVA, results are shown for time (T), group (G), and time × group (T × G) interactions. Values are presented as mean ± SD. *p < .05, significant interaction; #*p* < .05 vs. Pre within group.

### 3.2 Changes in blood lipids, obesity hormones and inflammation

**FIGURE 2:**
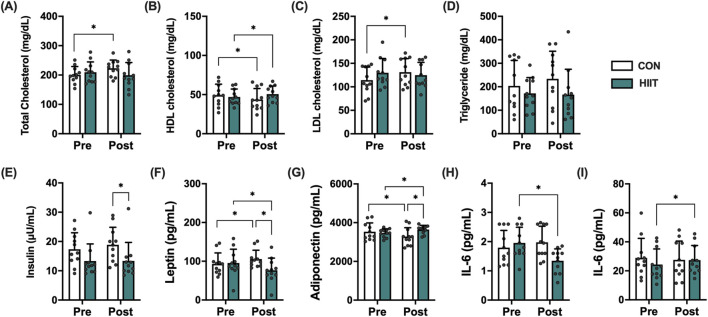
Effects of high-intensity interval training (HIIT) on metabolic and inflammatory markers in middle-aged men with obesity. Changes in **(A)** total cholesterol, **(B)** HDL cholesterol, **(C)** LDL cholesterol, **(D)** triglycerides, **(E)** insulin, **(F)** leptin, **(G)** adiponectin, **(H)** IL-6, and **(I)** IL-10 before (Pre) and after (Post) the 8-week intervention in the control (CON, white bars) and HIIT (black bars) groups. Data are presented as mean ± SD and individual data points for each participant are overlaid to visualize inter-individual variability in exercise responses. Statistical significance was determined using repeated-measures ANOVA with *post hoc* tests; *p* < 0.05 was considered statistically significant (* indicates significant difference).

Blood lipid profiles were analyzed to evaluate whether participation in the web-based HIIT program improved metabolic health in middle-aged men with obesity (Figures 2A–D). Significant group × time interactions were found for total cholesterol (F (1, 20) = 6.424, *p* = 0.020, ηp^2^ = 0.243), HDL-C (F (1, 20) = 23.832, *p* < 0.001, ηp^2^ = 0.544), and LDL-C (F (1, 20) = 12.629, *p* = 0.002, ηp^2^ = 0.387), indicating that lipid responses differed between groups. Simple-effects tests revealed that HDL-C significantly increased in the HIIT group but decreased in the CON group, while total cholesterol and LDL-C decreased only in the HIIT group. Triglycerides showed no significant interaction (F (1, 20) = 1.018, *p* = 0.325, ηp^2^ = 0.049). Obesity-related hormones also demonstrated robust group × time effects (Figures 2E–G): leptin (F (1, 20) = 78.011, *p* < 0.001, ηp^2^ = 0.796) and adiponectin (F (1, 20) = 68.594, *p* < 0.001, ηp^2^ = 0.774), whereas insulin did not (F (1, 20) = 2.291, *p* = 0.146, ηp^2^ = 0.103). Leptin markedly decreased in the HIIT group but increased in the CON group, while adiponectin increased in HIIT and decreased in CON, reflecting a favorable shift in adipokine profile. Regarding inflammatory markers (Figures 2H,I), IL-10 exhibited a significant group × time interaction (F (1, 20) = 4.713, *p* = 0.042, ηp^2^ = 0.191), suggesting an anti-inflammatory response following HIIT, whereas IL-6 (F (1, 20) = 0.455, *p* = 0.508, ηp^2^ = 0.022) showed no significant interactions. Collectively, these results indicate that real-time web-based HIIT elicited favorable alterations in lipid metabolism and adipokine balance characterized by increased HDL-C and adiponectin, reduced total cholesterol, LDL-C, and leptin and modestly enhanced the anti-inflammatory cytokine IL-10 compared with non-exercised controls.

### 3.3 Effects of web-based HIIT on aerobic capacity in middle-aged men with obesity

**FIGURE 3:**
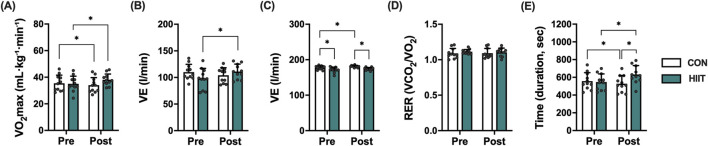
Effects of high-intensity interval training (HIIT) on cardiorespiratory fitness in middle-aged men with obesity. Changes in **(A)** maximal oxygen uptake (VO_2_max), **(B)** maximal ventilation (VE), **(C)** maximal heart rate (HRmax), **(D)** respiratory exchange ratio (RER), and **(E)** exercise time (duration) to exhaustion before (Pre) and after (Post) the 8-week intervention in the control (CON, white bars) and HIIT (black bars) groups. Data are expressed as mean ± SD and individual data points for each participant are overlaid to visualize inter-individual variability in exercise responses. Statistical significance was determined using repeated-measures ANOVA with *post hoc* tests. *P* < 0.05 was considered statistically significant (* indicates significant difference).

Aerobic capacity was evaluated to determine whether the web-based HIIT program improved cardiopulmonary function in middle-aged men with obesity (Figure 3). Significant group × time interactions were found for VO_2_max (mL·kg^-1^·min^-1^; F (1, 20) = 13.168, *p* = 0.002, ηp^2^ = 0.397), minute ventilation (VE; L·min^-1^; F (1, 20) = 7.900, *p* = 0.011, ηp^2^ = 0.283), and exercise duration (s; F (1, 20) = 42.575, *p* < 0.001, ηp^2^ = 0.680). VO_2_max increased in the HIIT group (35.00 → 38.06 mL kg^-1^·min^-1^) but declined slightly in the CON group (35.47 → 34.01). VE rose in HIIT (97.86 → 111.10 L min^-1^) but fell in CON (110.04 → 104.18), and exercise duration markedly increased in HIIT (547.27 → 631.82 s) while decreasing in CON (557.27 → 528.18). In contrast, maximal heart rate (beats·min^-1^) showed a time effect without interaction (F (1, 20) = 6.587, *p* = 0.018, ηp^2^ = 0.248), and the respiratory exchange ratio (RER) exhibited neither a time effect nor an interaction (F (1, 20) = 0.049, *p* = 0.828; interaction F (1, 20) = 0.235, *p* = 0.633). Collectively, these results indicate that real-time, web-based HIIT elicited favorable cardiopulmonary adaptations most notably increased VO_2_max, improved ventilatory response, and longer exercise tolerance relative to control over the 8-week period.

### 3.4 Functional gains in trunk strength and endurance induced by HIIT

Trunk muscle performance was evaluated to determine the effects of web-based HIIT on core stability and endurance (Table 4). A significant group × time interaction was observed for extension peak torque (EPT; F (1, 20) = 4.487, *p* = 0.047, ηp^2^ = 0.183), whereas flexion peak torque (FPT) showed no interaction (F (1, 20) = 0.568, *p* = 0.460, ηp^2^ = 0.028). Simple-effects analysis revealed a significant increase in EPT from pre-to post-intervention in the HIIT group (t (10) = −2.974, *p* = 0.014, *d* = 0.90), while no significant change was noted in the control group. For average power variables, extension average power (EAP) demonstrated a robust group × time interaction (F (1, 20) = 17.560, *p* = 0.001, ηp^2^ = 0.468), whereas flexion average power (FAP) did not (F (1, 20) = 2.316, *p* = 0.144, ηp^2^ = 0.104). Simple-effects testing indicated that EAP significantly increased over time in the HIIT group (t (10) = −4.330, *p* = 0.002, *d* = 1.30), suggesting improved dynamic endurance of trunk extensors. Collectively, these findings indicate that participation in the real-time, web-based HIIT program elicited significant improvements in trunk extensor strength and endurance, which are critical components of core stability, postural control, and overall functional performance in middle-aged men with obesity.

**TABLE 4:** Functional gains in trunk strength and endurance induced by HIIT.

Muscle function variable	Time point	CON (n = 11)	HIIT (n = 11)	Source of variation	F-statistic	*p*-value
Extensor peak torque (60°, %BW)	Pre Post	283.55 ± 18.74 283.45 ± 14.86	250.09 ± 14.57 274.27 ± 16.12^#^	T	4.42	0.048^*^
				G	0.929	0.365
				T × G	4.487	0.047^*^
Flexors peak torque (60°, %BW)	Pre Post	216.73 ± 14.32 212.45 ± 17.88	202.73 ± 17.70 214.55 ± 9.95	T	0.125	0.727
				G	0.1	0.755
				T × G	0.568	0.46
Extensor average power (240°, %BW)	Pre Post	265.18 ± 33.13 241.91 ± 27.44	231.64 ± 22.63 301.73 ± 20.90^#^	T	4.416	0.048^*^
				G	0.135	0.717
				T × G	17.56	0.001^*^
Flexors average power (240°, %BW)	Pre Post	212.55 ± 23.87 180.91 ± 19.97	199.00 ± 23.72 210.55 ± 23.20	T	0.501	0.487
				G	0.078	0.783
				T × G	2.316	0.144

Two-way repeated-measures ANOVA, results are shown for time (T), group (G), and time × group (T × G) interactions. Values are presented as mean ± SD. *p < .05, significant main effect or interaction; #*p* < .05 vs. Pre within group.

### 3.5 Effects of web-based HIIT on lower extremity strength and muscular endurance

Lower-extremity (quadriceps and hamstring) strength and endurance were assessed to determine whether the HIIT intervention altered functional performance in middle-aged men with obesity (Table 5). For peak torque, significant group × time interactions were observed for right-leg extension (EPTR: F (1, 20) = 7.768, *p* = 0.011, ηp^2^ = 0.280), left-leg extension (EPTL: F (1, 20) = 4.628, *p* = 0.044, ηp^2^ = 0.188), right-leg flexion (FPTR: F (1, 20) = 13.752, *p* = 0.001, ηp^2^ = 0.407), and left-leg flexion (FPTL: F (1, 20) = 8.297, *p* = 0.009, ηp^2^ = 0.293). Simple-effects tests indicated that EPTR decreased over time in the CON group (t (10) = 3.623, *p* = 0.005, *d* = 1.09), whereas flexion torque increased significantly in the HIIT group (FPTR: t (10) = −3.949, *p* = 0.003, *d* = 1.19; FPTL: t (10) = −3.497, *p* = 0.006, *d* = 1.05). Baseline between-group differences were present for FPTR (pre: t (20) = 3.154, *p* = 0.005) and FPTL (pre: t (20) = 2.751, *p* = 0.012), consistent with these interactions.

**TABLE 5:** Effects of web-based HIIT on lower extremity strength and muscular endurance.

Muscle function variable	Time point	CON (n = 11)	HIIT (n = 11)	Source of variation	F-statistic	*p*-value
Extensor peak torque right (60°, %BW)	Pre Post	195.09 ± 16.08 173.64 ± 12.55^#^	194.82 ± 11.41 195.91 ± 8.15	T	6.337	0.020^*^
				G	0.417	0.526
				T × G	7.768	0.011^*^
Extensor peak torque left (60°, %BW)	Pre Post	192.36 ± 18.26 172.36 ± 12.35	184.18 ± 6.88 189.45 ± 6.52	T	1.571	0.224
				G	0.078	0.783
				T × G	4.628	0.044^*^
Flexors peak torque right (60°, %BW)	Pre Post	108.55 ± 10.57 102.64 ± 9.41	72.45 ± 4.39 95.45 ± 7.09^#^	T	4.806	0.040^*^
				G	3.913	0.062
				T × G	13.752	0.001^*^
Flexors peak torque left (60°, %BW)	Pre Post	109.09 ± 11.59 101.82 ± 9.32	74.73 ± 4.67 89.27 ± 5.87^#^	T	0.922	0.348
				G	4.425	0.048^*^
				T × G	8.297	0.009^*^
Extensor average power right (240°, %BW)	Pre Post	162.36 ± 14.17 151.73 ± 14.03	135.73 ± 11.91 141.45 ± 11.60	T	0.199	0.660
				G	1.110	0.305
				T × G	2.211	0.153
Extensor average power left (240°, %BW)	Pre Post	153.64 ± 15.68 140.36 ± 10.92	129.82 ± 8.01 135.36 ± 10.50	T	0.778	0.388
				G	0.829	0.374
				T × G	4.616	0.044^*^
Flexors average power right (240°, %BW)	Pre Post	84.36 ± 8.85 83.36 ± 9.00	53.00 ± 2.42 69.36 ± 6.39^#^	T	3.115	0.093
				G	6.115	0.023^*^
				T × G	3.979	0.060
Flexors average power left (240°, %BW)	Pre† Post	93.64 ± 9.11 80.36 ± 7.09^#^	58.45 ± 5.02 64.18 ± 4.89	T	1.388	0.253
				G	8.151	0.010^*^
				T × G	8.800	0.008^*^

Two-way repeated-measures ANOVA, results are shown for time (T), group (G), and time × group (T × G) interactions. Values are presented as mean ± SD. *p < .05, significant main effect or interaction; #*p* < .05 vs. Pre within group; †*p* < .05 vs. CON, at the same time point.

For muscular endurance (average power at 240°·s^-1^), right-leg extension (EAPR: F (1, 20) = 2.211, *p* = 0.153) and right-leg flexion (FAPR: F (1, 20) = 3.979, *p* = 0.060) showed no significant interactions, whereas left-leg endurance exhibited significant interactions for extension (EAPL: F (1, 20) = 4.616, *p* = 0.044, ηp^2^ = 0.188) and flexion (FAPL: F (1, 20) = 8.800, *p* = 0.008, ηp^2^ = 0.306). Simple-effects tests showed a time-related increase in right-leg flexion power within the HIIT group (t (10) = −2.695, *p* = 0.022, *d* = 0.81) and a decrease in left-leg flexion power within the CON group (t (10) = −2.381, *p* = 0.039, *d* = 0.72). Baseline between-group differences were also present for FAPR (pre: t (20) = 3.419, p = 0.003) and FAPL (pre: t (20) = 3.383, *p* = 0.003). Taken together, lower-extremity outcomes displayed domain-specific, group-dependent trajectories. Peak torque changes differed between groups (notably larger interaction effects for flexion torque; ηp^2^ = 0.29–0.41), and endurance outcomes showed side-specific interactions (left > right; ηp^2^ = 0.19–0.31). These patterns indicate differential adaptations of knee musculature across groups over the 8-week period, rather than uniform improvement in all indices.

## 4 Discussion

Obesity is a major determinant of COVID-19 severity and mortality, primarily due to its association with systemic inflammation, impaired metabolic regulation, and cardiopulmonary dysfunction. Sedentary lifestyles imposed by pandemic restrictions have further accelerated weight gain, visceral fat accumulation, and muscle loss in men, thereby compounding vulnerability to metabolic and infectious diseases. Consistent with previous findings that HIIT reduces BMI, WHR, and visceral adiposity during the COVID-19 pandemic (da Silveira et al., 2021; Khalafi and Symonds, 2021), the present study demonstrated quantitatively meaningful improvements following participation in a non-face-to-face (web-based) HIIT program. Specifically, fat mass decreased by 6.6% (−2.01 kg; *d* = 1.38) without significant changes in total body weight or lean mass, indicating a selective reduction in adiposity rather than overall weight loss. Aerobic capacity improved substantially, with VO_2_max increasing by 3.06 mL kg^-1^·min^-1^ (∼8.7%), accompanied by rises in minute ventilation (+13.24 L min^-1^) and exercise duration (+84.6 s, ∼15.4%) in the HIIT group, while the control group showed slight declines. Lipid metabolism also improved, with HDL-C increasing and total/LDL cholesterol decreasing (ηp^2^ = 0.24–0.54), alongside favorable hormonal and inflammatory changes leptin decreased and adiponectin increased (ηp^2^ = 0.77–0.80), and IL-10 increased (ηp^2^ = 0.19) reflecting an anti-inflammatory shift. Functionally, trunk extensor strength rose by 9.7% (*d* = 0.90) and extensor average power by 30.2% (*d* = 1.30), while lower-limb flexor strength increased by 31.8% (right) and 19.5% (left) in the HIIT group. Together, these magnitudes underscore that real-time, web-based HIIT effectively enhances metabolic, cardiopulmonary, and neuromuscular functions in men with obesity.

However, no significant changes were observed in total body weight or lean mass. This may reflect the nature of body-weight-based HIIT, in which participants use their own body mass as resistance. Such exercise predominantly induces fat loss and metabolic improvements rather than hypertrophic adaptations, especially over short intervention periods (Lopez et al., 2022; Prieto González and Sedlacek, 2021). Therefore, the maintenance of lean tissue alongside reduced fat mass indicates a favorable alteration in body composition rather than a mere decrease in body weight. These findings indicate that remotely supervised exercise interventions can elicit adaptations comparable to those achieved through conventional face-to-face modalities. Moreover, when energy expenditure is matched, HIIT has been shown to induce greater reductions in body weight and fat ratio compared with moderate-intensity continuous training (MICT) (Andreato et al., 2019), underscoring its practicality as a time-efficient intervention for men, particularly those with sedentary occupations or a preference for dynamic exercise modalities (Sanchis-Gomar et al., 2021).

COVID-19 severity has been strongly linked to comorbidities such as diabetes, hypertension, and dyslipidemia. Previous studies have reported that HIIT lowers fasting glucose and improves insulin sensitivity in adults with obesity (Ryan et al., 2020), mitigates insulin resistance in sedentary older populations (Hayes et al., 2020), and enhances glycemic control in women (Dashti et al., 2021). In line with these findings, the present study confirmed the metabolic benefits of web-based HIIT, particularly through improvements in lipid profiles. Whereas short-term quarantine has been associated with increases in total cholesterol and LDL cholesterol and decreases in HDL cholesterol (Perrone et al., 2021), participants in the HIIT group demonstrated elevated HDL cholesterol levels, a clinically meaningful adaptation given that LDL cholesterol is directly implicated in atherosclerosis and ischemic heart disease and has been linked to COVID-19 mortality (Giraldi et al., 2020). Notably, Bowden Davies et al., 2018 reported that 2 weeks of physical inactivity can impair cardiac function and insulin sensitivity, reinforcing the significance of HDL improvements observed in this study for cardiovascular protection during pandemic-related restrictions (Bowden Davies et al., 2018). Although minor changes in certain hormonal markers were observed in the control group, these changes were not statistically significant and may be attributable to natural biological variability, expectation bias, or subtle lifestyle modifications. In contrast, the HIIT group exhibited a favorable hormonal profile characterized by reduced insulin and leptin levels (both typically elevated in obesity) and increased adiponectin concentrations, reflecting improved insulin sensitivity, leptin responsiveness, and adipose tissue function. Together with the increases in HDL cholesterol and IL-10 and the reductions in LDL cholesterol and IL-6, these adaptations highlight the comprehensive cardiometabolic and anti-inflammatory benefits of web-based HIIT in middle-aged men with obesity.

Visceral adiposity also drives pro-inflammatory cytokine production, contributing to severe outcomes in COVID-19. Elevated IL-6, IL-1β, and TNF-α levels amplify immune activation, damaging pulmonary structures and worsening disease severity (Jose and Manuel, 2020). Exercise interventions, including HIIT, have been reported to enhance anti-inflammatory responses and reduce cytokine expression (Stebbing et al., 2020). Our findings corroborate this evidence, showing that 8 weeks of web-based HIIT decreased IL-6 and increased IL-10. These immunomodulatory adaptations are of clinical importance, as they may reduce the risk of cytokine storms and subsequent complications in obese individuals with COVID-19.

Hormonal adaptations further underscore the value of HIIT for regulating obesity-related inflammation. Elevated leptin promotes neutrophil and T-cell proliferation and drives IL-6 production (Larsson et al., 2021; Maurya et al., 2021). Conversely, adiponectin exerts anti-inflammatory effects by stimulating IL-10 and IL-1RA production, but its levels are typically reduced in obesity (Vyas, 2021). Conversely, adiponectin exerts anti-inflammatory effects by stimulating IL-10 and IL-1RA production, but its levels are typically reduced in obesity (Maurya et al., 2021). Previous studies have demonstrated that HIIT increases adiponectin and lowers the leptin/adiponectin ratio in adolescents with obesity (Khanevari et al., 2021) and reduces leptin resistance more effectively than MICT in overweight cancer survivors (Hooshmand Moghadam et al., 2021). In line with these findings, our study revealed decreased leptin and increased adiponectin levels after HIIT. Similar results have been observed in overweight female students, in whom HIIT significantly elevated adiponectin and produced strong anti-inflammatory effects (Hovsepian et al., 2021). Collectively, these hormonal changes highlight the capacity of HIIT to regulate obesity-related inflammation and to mitigate the severity of COVID-19 outcomes.

Younger and healthier people recover faster from the symptoms of COVID-19, whereas the elderly have a higher mortality rate. Cardiorespiratory fitness, reflected by VO_2_max, is another critical determinant of resilience against COVID-19. Lower VO_2_max has been identified as a predictor of poor clinical prognosis (Sallis et al., 2021). Mechanistically, obesity increases angiotensin-converting enzyme 2 (ACE2) expression in adipose tissue, thereby facilitating viral entry (Maurya et al., 2021), while VO_2_max is inversely associated with ACE2 activity. Previous studies have consistently shown that HIIT improves cardiopulmonary function, with 6 weeks of training enhancing VO2max and peak power output in healthy adults (Marterer et al., 2020), and 12 weeks producing superior improvements in cardiovascular risk markers compared with MICT. The present findings, which revealed significant improvements in VO_2_max in middle-aged men with obesity following web-based HIIT, provide further evidence that real-time remote interventions can preserve aerobic capacity and immune competence during pandemic conditions. Although a learning or expectation bias associated with repeated VO_2_max testing cannot be completely excluded, the control group did not exhibit a statistically significant increase in VO_2_max. Therefore, the significant between-group difference suggests that the observed improvement in aerobic capacity was primarily attributable to the HIIT intervention rather than procedural familiarization or motivational effects. This interpretation is consistent with previous findings showing that repeated VO_2_max tests can yield small improvements due to learning effects or reduced anxiety, but such changes are typically non-significant in non-training populations (Midgley and Carroll, 2009).

Skeletal muscle is also a key regulator of metabolic and immune health. Greater muscle mass supports vascular function and reduces the risk of severe COVID-19 (Denay et al., 2020), whereas prolonged sedentary behavior promotes muscle atrophy and impairs functional performance. Studies have shown that 2 weeks of bed rest can reduce muscle mass by 6%–8% (0.4%–0.6%/day) and significantly impair contraction capacity. Conversely, Miyamoto-Mikami et al., 2018 reported that interval training such as Tabata increased thigh circumference by 3% ± 1% and lower extremity muscle strength by 10% ± 3% (Miyamoto-Mikami et al., 2018). In line with these findings, our study demonstrated significant improvements in both upper- and lower-limb strength after 8 weeks of web-based HIIT. These adaptations may reflect enhanced protein synthesis, improve mitochondrial function, and reduce systemic inflammation, collectively supporting the maintenance of muscular performance during prolonged isolation. The present findings further align with recent evidence demonstrating that video-guided HIIT enhances exercise adherence, technique accuracy, and safety through real-time visual supervision (Bellissimo et al., 2025). This highlights the importance of guided feedback in optimizing the outcomes of remote training interventions.

Despite several limitations, this study provides meaningful preliminary evidence supporting the efficacy of web-based HIIT during pandemic-related restrictions. First, the relatively small sample size may limit the generalizability of the findings. Second, although dietary intake was tracked *via* daily food diaries verified by a registered dietitian, nutritional control was not fully standardized. This variability in dietary intake could have influenced hormonal responses, such as changes in insulin, cortisol, or inflammatory cytokines, which are sensitive to short-term energy balance and macronutrient composition. While strict dietary regulation may enhance internal validity, it would have been impractical and potentially burdensome under quarantine conditions. Additionally, a modest difference in mean age between groups (approximately 5 years) could have introduced minor variability in physiological responses. However, random allocation and the within-subject repeated-measures design likely minimized its confounding influence. Given the exploratory nature and limited sample size of this pilot trial, age was not included as a covariate to avoid statistical overfitting. Future studies with larger and more diverse cohorts should consider incorporating age and other demographic factors as covariates to further control for potential bias. Future studies should include larger cohorts and standardized dietary protocols to strengthen external validity and confirm the present findings.

In conclusion, this pilot study provides preliminary evidence that high-intensity BW-IT, delivered in a non-face-to-face (web-based) and real-time format, may improve body composition, cardiometabolic markers, inflammatory profiles, aerobic capacity, and muscular performance in men with obesity. These findings suggest that remotely delivered, interactive BW-IT programs could serve as feasible and accessible strategies to support metabolic health, enhance immune resilience, and help maintain overall wellbeing during periods of restricted in-person activity. Furthermore, such approaches may hold potential as scalable components of future public health initiatives, particularly under conditions of social distancing or infectious disease outbreaks.

## Data Availability

The original contributions presented in the study are included in the article/supplementary material, further inquiries can be directed to the corresponding authors.

## Abbreviations

HIIT, high-intensity interval training; MICT, moderate-intensity continuous training; BMI, body mass index; BMD, bone mineral density; VO2max, maximal oxygen uptake; HRmax, maximal heart rate; VE, minute ventilation; RER, respiratory exchange ratio; IL, interleukin; CRP, C-reactive protein; RCT, randomized controlled trial; ACSM, American College of Sports Medicine; WHO, World Health Organization; RPE, rating of perceived exertion.
